# Chronic Elevation of Systemic Glucagon-Like Peptide-1 Following Surgical Weight Loss: Association with Nausea and Vomiting and Effects on Adipokines

**DOI:** 10.1007/s11695-014-1507-4

**Published:** 2014-11-20

**Authors:** Noora Al-Rasheid, Rosaire Gray, Pratik Sufi, Nephtali Marina-Gonzalez, Mohammed Al-Sayrafi, Elizabeth Atherton, Vidya Mohamed-Ali

**Affiliations:** 1 Adipokines & Metabolism Research Group, Centre for Clinical Pharmacology, Division of Medicine, University College London, 5 University St., London, WC1E 6JJ UK; 2 North London Obesity Surgery Service, Whittington Hospital, London, UK; 3 Life Science Research Division, Anti Doping Laboratory Qatar (ADLQ), Doha, Qatar

**Keywords:** GLP-1, Nausea, Adipokines

## Abstract

We determined whether persistent nausea and vomiting (N/V) symptoms following Roux-en-Y gastric bypass surgery is due to elevated systemic glucagon-like peptide-1 (GLP-1) and leptin in female non-diabetic subjects. Subjects with N/V post-Roux-en-Y gastric bypass (RYGB) surgery had significantly elevated fasting GLP-1 levels compared to that with post-operative asymptomatic subjects and to morbidly obese, obese and lean subjects not undergoing surgery. Weight loss, glycaemia, insulin and post-prandial GLP-1 levels were similar in all post-operative subjects. Despite comparable BMI, leptin was significantly lower in symptomatic subjects. Furthermore, leptin secretion from subcutaneous adipose tissue was inhibited by GLP-1 (0.1–1.0 nM; *n* = 6). Persistent N/V following RYGB surgery is associated with elevated fasting GLP-1, but lower leptin levels. The latter may be a consequence of the direct GLP-1 inhibition of leptin secretion from adipose tissue*.*

## Introduction

Bariatric surgery is to date the only intervention that results in significant and sustained weight loss for morbid obesity [1]. Procedures such as Roux-en-Y gastric bypass (RYGB), which combines gastric restriction with the bypass of the stomach and proximal intestine, are more effective than other forms of metabolic surgery [1]. However, a proportion of patients develops sustained and debilitating nausea and vomiting (N/V) symptoms after RYGB as well as with other procedures such as sleeve gastrectomy that is not related to mechanical obstruction [2, 3].

Glucagon-like peptide-1 (GLP-1) is an incretin hormone synthesised and secreted by endocrine L cells in the small intestine and centrally by neurons in the nucleus tractus solitarius (NTS) of the brainstem. Under physiological conditions GLP-1 augments insulin secretion after nutrient ingestion and decelerates gastric emptying and inhibits intestinal transit time [4]. GLP-1 infusions reduce energy intake in lean and overweight subjects [4]. Endogenous GLP-1 appears to reduce food intake by acting in a paracrine-like fashion on adjacent GLP-1 receptors (GLP-1R) expressed on vagal afferents innervating the GI tract, rather than acting directly on GLP-1R in the brain [4].

A recognised side effect of GLP-1 analogues used to treat diabetes is nausea, which occurs in up to 50 % of patients [5]. Studies in rats suggest that the nausea-inducing effects of peripheral exendin-4, a GLP-1R agonist, are mediated by a vagal-independent pathway that appears to involve the blood brain barrier (BBB) penetrance and subsequent GLP-1R activation in the central nervous system (CNS), most likely in the NTS [6]. Post-prandial GLP-1 plasma levels are significantly increased following RYGB and remain elevated for at least 1 year [7]. Increased GLP-1 levels are associated with enhanced satiety and are hypothesised to be related to decreased gastrointestinal transit time in bypass patients rather than weight loss alone [8].

There is also data suggesting a relationship between leptin and symptoms of severe, but not mild-moderate, nausea in women during pregnancy [9, 10]. Furthermore, the most common side effect of treatment with metreleptin is nausea [11]. Thus, whether leptin may provoke N/V in a subset of women following RYGB has not been investigated. The aim of this study was to test whether increased GLP-1 and leptin levels after surgery could be a contributing factor to the persistent N/V. We compared post-absorptive and post-prandial levels of GLP-1 and leptin between asymptomatic subjects and patients with persistent N/V following RYGB and between lean, overweight and obese subjects not scheduled for surgery. In addition, we determined the possibility of cross-talk between the gut peptide and adipokines, by investigating the effect of GLP-1 on leptin secretion in vitro.

## Subjects and Methods

### Subjects

Five groups of subjects were recruited; obese subjects with persistent and prolonged N/V after RYGB (group 1: *n* = 10), asymptomatic post-operative obese subjects (group 2: *n* = 10), morbidly obese subjects (group 3: *n* = 7), overweight/obese subjects (group 4: *n* = 6) and lean healthy subjects (group 5: *n* = 9). Subjects in group 1 had upper gastrointestinal tract endoscopy and imaging to exclude an obstructive cause for their symptoms. The subject characteristics are shown in Table 1. Exclusion criteria were current treatment with warfarin or non-steroidal anti-inflammatory drugs (NSAIDs), type 2 diabetes mellitus (T2DM) and malignancy or substance abuse. The study was approved by the National Research Ethics Committee, and written informed consent was obtained from all participants.

**Table 1:** Subject characteristics (in vivo study)

	Group 1 N/V (*n* = 10)	Group 2 No N/V (*n* = 10)	Group 3 MO (*n* = 7)	Group 4 OW (*n* = 6)	Group 5 Lean (*n* = 9)
Age (years)	41.4 ± 3.4	38 ± 3.1	39.3 ± 4.3	45.5 ± 3.9	34.7 ± 4.6
BMI (kg/m^2^)	30.6 ± 2.3	31.2 ± 2.0	46.3 ± 1.7	31.8 ± 1.9	21.3 ± 0.7
SBP (mmHg)	119 ± 3	109 ± 3	127 ± 7	121 ± 12	21.3 ± 1
DBP (mmHg)	73 ± 5	72 ± 3	81 ± 4	79 ± 2	76.6 ± 12
Fasting glucose (mmol/l)	5.0 ± 2.4	4.5 ± 0.5	5.07 ± 0.9	5.2 ± 0.7	4.7 ± 0.5
Fasting insulin (mIU/l)	5.0 ± 2.4	4.5 ± 1.5	10.2 ± 7.5	9.4 ± 4.1	4.1 ± 2
HOMA-IR	1.2 ± 0.6	0.9 ± 0.3	2.4 ± 2.2*	2.1 ± 0.8*	0.8 ± 0.4
Total cholesterol (mmol/l)	3.5 ± 0.35	4.1 ± 0.12	4.0 ± 0.43	4.4 ± 0.26	4.9 ± 0.38
HDL cholesterol (mmol/l)	1.2 ± 0.12	1.4 ± 0.12	0.99 ± 0.07	1.3 ± 0.14	1.9 ± 0.29**
LDL cholesterol (mmol/l)	1.8 ± 0.29	2.4 ± 0.25	2.5 ± 0.41	2.7 ± 0.23	2.6 ± 0.29
Triglycerides (mmol/l)	1.1 ± 0.23	0.85 ± 0.05	1.1 ± 0.18	0.81 ± 0.24	0.81 ± 0.13

Data are expressed as mean ± standard deviation

*BMI* body mass index, *SBP* systolic blood pressure, *DBP* diastolic blood pressure, *HOMA-IR* homeostatic model assessment-insulin resistance, *HDL* high-density lipoprotein, *LDL* low-density lipoprotein. *N/V* nausea and vomiting, *MO* morbidly obese, *OW* obese and overweight

*p < 0.05 compared to the post-surgery and lean groups; **p < 0.05 comparing the lean to all other groups

Subjects attended the hospital outpatient clinic in the morning after an overnight fast. The post-operative patients were studied 18.8 ± 3.0 months after surgery. Fasting blood samples were collected for measurement of plasma glucose, lipids, insulin, leptin, adiponectin and GLP-1. Subjects consumed a standard 180 kcal meal (9.6 g of fat, 44.3 g of carbohydrate and 8.5 g of protein), and additional blood samples for GLP-1, glucose and insulin were collected at 45, 120 and 180 min after the meal. The meal size was determined by subjects with post-operative N/V unable to tolerate larger portions.

### Anthropometric Measurements

BMI was calculated as the weight (kg) divided by the square of the height (m^2^). Blood pressure was measured with a digital device (Omron, Netherlands).

### Assays

Plasma glucose, serum triglycerides, total, low-density lipoprotein (LDL) and high-density lipoprotein (HDL) cholesterol concentrations were determined with commercial reagents. Glucose and lipid assays were performed in the pathology laboratories at the Whittington Hospital using routine procedures. GLP-1 levels were measured by ELISA specific for the active form of GLP-1 (7–36). Leptin, adiponectin and insulin levels were determined using commercially available ELISA. Insulin resistance (IR) was calculated by the homeostatic model assessment (HOMA) using: HOMA-IR = (glucose in mmol/l × insulin in mIU/l)/22.5.

### In Vitro Study

Abdominal subcutaneous adipose tissue (SAT) samples were obtained from six morbidly obese, non-diabetic patients undergoing bariatric surgery (mean age 47.6 ± 9.9 years, BMI 43.6 ± 8.0 kg/m^2^) for use in organ cultures. SAT organ cultures (0.05 mg/500 μl) were incubated in serum-free media with 1 % penicillin/streptomycin solution containing recombinant human GLP-1 (0, 0.1, 0.5 and 1.0 nM diluted in phosphate-buffered saline with 0.1 % bovine serum albumin). After 4 or 16 h of incubation (37 °C and 5 % CO_2_) culture media were collected, and leptin levels were determined.

### Statistical Analysis

Results are expressed as mean and standard deviation for normally distributed data and median and interquartile ranges for skewed data. Significance was defined as *P* < 0.05.

## Results

**Fig. 1:**
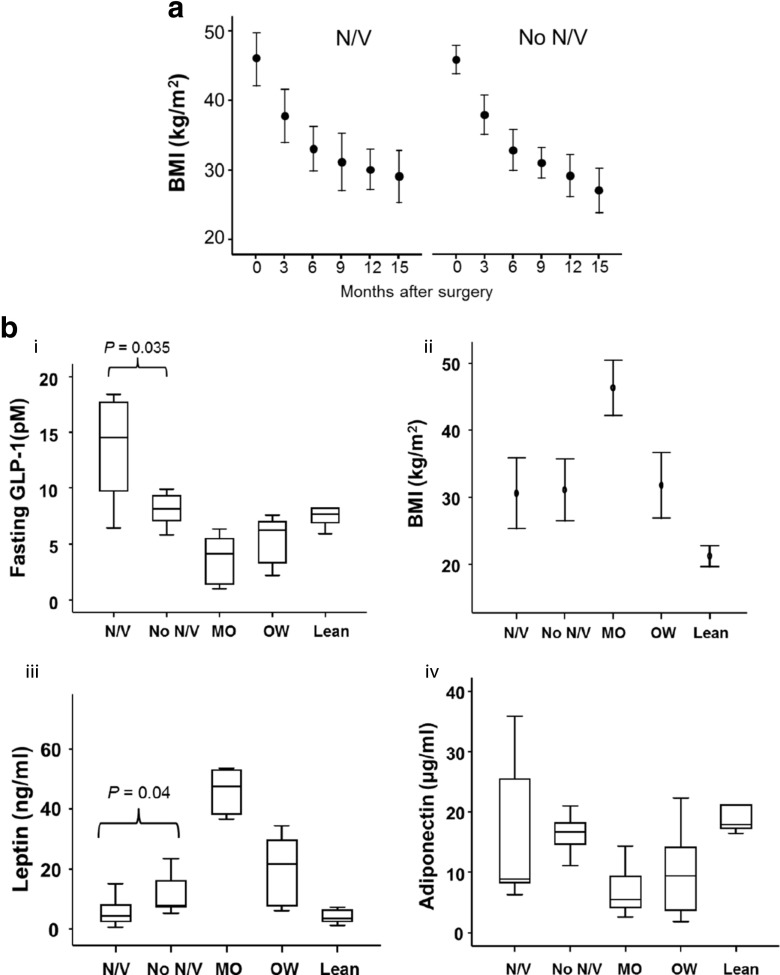
**a** Weight loss following Roux-en-Y gastric bypass in subjects with and without persistent nausea and vomiting symptoms. BMI of patients with and without persistent N/V symptoms was recorded for 15 months following RYGB (*n* = 10 per group). There were no significant differences in the change in BMI over time between the two groups. Data are shown as mean and standard deviation and comparisons carried out by *t* test. *N/V* nausea and vomiting, *BMI* body mass index, *RYGB* Roux-en-Y gastric bypass. **b** Changes in fasting levels of GLP-1 and adipokines. *i* Fasting plasma GLP-1 levels. In subjects with persistent N/V, fasting GLP-1 levels were elevated (*P* = 0.035) compared to that in subjects without N/V, morbidly obese subjects, obese and overweight subjects and lean subjects. Data are shown as median and interquartile ranges and comparisons made by Mann Whitney *U* test. *ii* and *iii* BMI and fasting systemic leptin levels. Although BMI (*ii*) were similar between N/V and non N/V groups, plasma leptin levels (*iii*) were significantly lower in N/V groups compared to non N/V groups (*P* = 0.04). Data in *ii* are shown as mean (SD) and in iii as median (interquartile range). *iv* Fasting plasma adiponectin levels. Plasma adiponectin was not significantly different between N/V and non N/V groups. Data are shown as median (interquartile range) and comparisons made by Mann Whitney *U* test. Groups: post-operative nausea and vomiting (N/V) *n* = 10; post-operative non N/V *n* = 10; morbidly obese (MO) *n* = 7; obese and overweight (OW) *n* = 6; and lean *n* = 9

All subjects were female, non-diabetic, normotensive and normolipidaemic. The groups were matched for age. BMI on the day of study varied according to the group (Table 1). The mean BMI of morbidly obese patients and lean subjects were 46.3 and 21.3 kg/m^2^, respectively. The BMI of the post-operative and the obese/overweight patients was not significantly different. Weight loss was not significantly different in subjects with and without N/V (Fig. 1a) 15 months after RYGB surgery. Fasting plasma GLP-1 levels were significantly higher in the subjects with persistent N/V post-RYGB surgery compared to all other groups (*P* = 0.007, Fig. 1b(i)). This difference was significant (*P* = 0.03) despite similar BMI (*P* = 0.86) in the two post-operative groups.

**Fig. 2:**
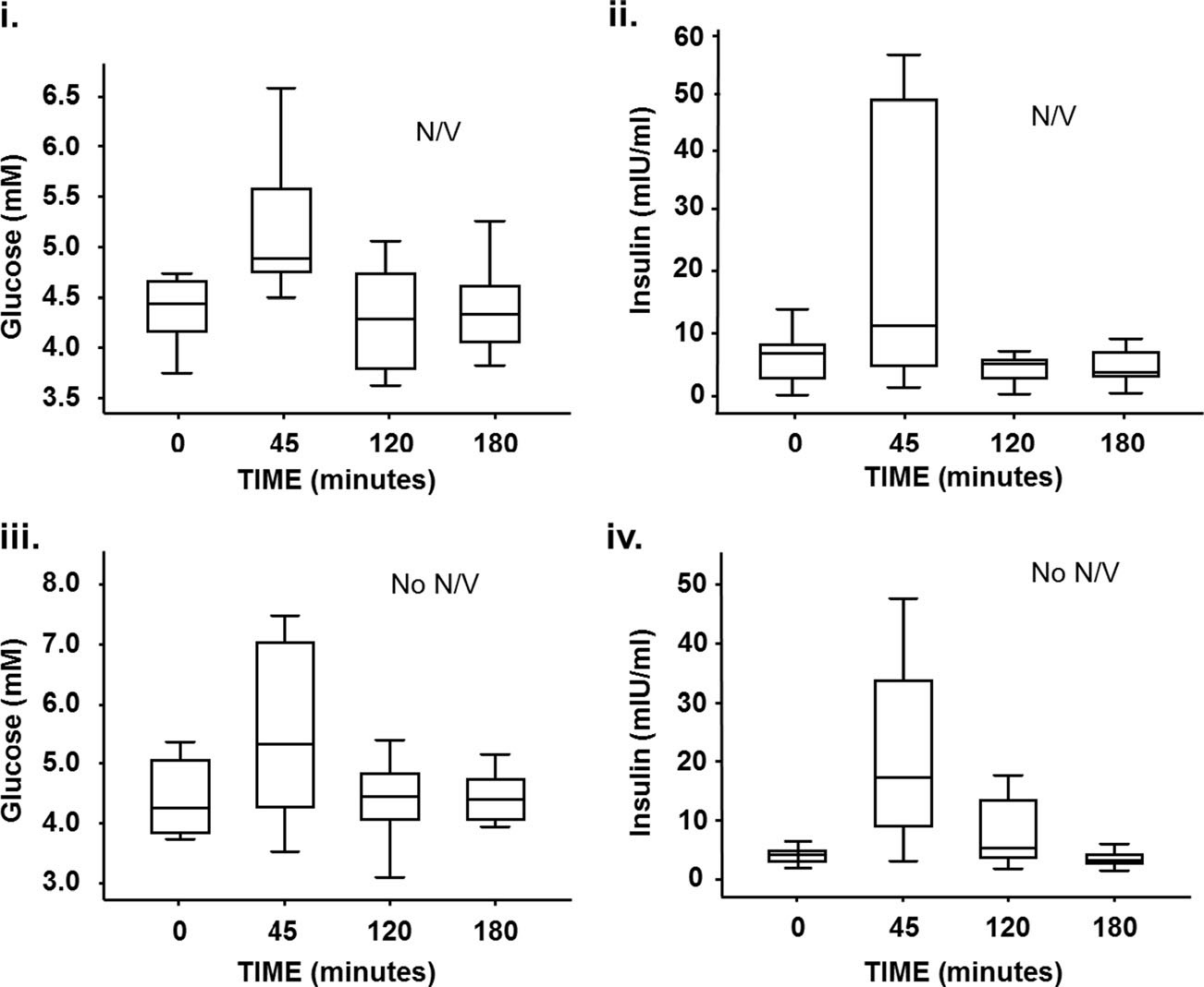
Glucose and insulin response to a meal challenge in subjects with and without nausea and vomiting post Roux-en-Y gastric bypass surgery. Following an 180-kcal meal challenge, the glucose and insulin responses were similar in subjects with (*i* and *ii*) and without N/V symptoms (*iii* and *iv*) after Roux-en-Y gastric bypass surgery. Data are shown as median and interquartile ranges and comparisons made with Mann Whitney *U* test

Morbidly obese subjects had the highest systemic leptin levels. Subjects with post-operative N/V had significantly lower plasma leptin concentrations compared to that of asymptomatic post-operative patients. In fact, leptin levels in these patients were similar to those observed in lean subjects (Fig. 1b(ii and iii)). However, fasting plasma adiponectin levels were not significantly different in subjects with post-operative N/V compared with all other groups (*P* = 0.2 Fig. 1b(iv)). Furthermore, there was no significant difference in the post-prandial glucose and insulin response in subjects with and without post-operative N/V (Fig. 2). No significant differences in the magnitude of the post-prandial GLP-1 response were seen in subjects with and without post-operative N/V.

In vitro experiments showed that leptin secretion from SAT was significantly suppressed in the presence of GLP-1 after 16 h [GLP-1 treatment: 0 vs. 0.1 nM {2.6 ng/ml (0.4) vs. 1.9 ng/ml (0.4), *P* = 0.06}; 0 vs. 0.5 nM {2.6 ng/ml (0.4) vs. 1.1 ng/ml (0.31), *P* = 0.003}; 0 vs. 1.0 nM {2.6 ng/ml (0.4) vs. 1.0 ng/ml (0.29), *P* = 0.001}]. Treatment with GLP-1 for 4 h did not produce any significant changes in SAT leptin release.

## Discussion

N/V is a common side effect experienced by the majority of patients undergoing RYGB, but symptoms usually disappear shortly after the operation [12]. However, approximately 1–5 % of patients present with difficult to control persistent N/V despite the absence of mechanical abnormalities [2]. We found that symptomatic patients have significantly higher basal, but not post-prandial, GLP-1 levels, suggesting that non-mechanical chronic N/V symptoms after RYGB surgery may be due, at least in part, to chronically elevated GLP-1 levels. Increased GLP-1 concentrations may therefore also explain similar symptoms seen after other bariatric procedures such as sleeve gastrectomy [13]. The significant number of diabetic patients treated with exendin-4 also experience N/V [6], providing further support for the role of elevated GLP-1 levels on the generation of symptoms.

Exendin-4 induces nausea by penetrating the BBB and subsequently activating GLP-1R in the medial NTS [5, 4]. Whether GLP-1 induces N/V by direct actions on the NTS or indirectly through vagal afferent pathway is not known. However, endogenous GLP-1 has a very short half-life and is rapidly degraded by DPP-4 enzyme, which makes it unlikely to cross the BBB.

Despite higher basal GLP-1 levels in symptomatic patients compared to those without symptoms post-operatively, weight loss, insulin sensitivity and adiponectin levels were not significantly different in both groups. Thus, the beneficial effects of RYGB on improving insulin sensitivity and weight loss were not affected by elevated basal GLP-1 levels and the symptoms of N/V. Contrary to data from the persistent N/V that accompanies pregnancy [10], systemic leptin levels were lower in the symptomatic compared to asymptomatic subjects, despite similar post-operative BMI. Our in vitro study showed that chronic (16 h), but not acute (4 h), exposure to GLP-1 inhibited leptin secretion from human subcutaneous adipose tissue. GLP-1 has been shown to inhibit visfatin and exendin-4 to stimulate adiponectin secretion from 3T3-L1 adipocytes [14, 15]. The acute administration of synthetic human GLP-1 to obese patients with and without T2DM reduced circulating interleukin-6 in only those with T2DM, without affecting levels of leptin, adiponectin or obestatin [16]. Therefore, it appears that only chronic, but not acute, exposure to elevated levels of GLP-1, either in vivo or in vitro, leads to inhibition of leptin.

Leptin stimulates GLP-1 secretion from the hypothalamus and may be involved in the regulatory mechanisms of GLP-1 production by L cells [17]. As leptin stimulates GLP-1 secretion in a negative feedback mechanism, GLP-1 may directly inhibit leptin secretion. Inhibition of leptin secretion by GLP-1 was observed in subcutaneous adipose tissue, the major depot contributing to its systemic levels. That this is a direct effect on secretion rather than a reflection of differences in fat mass in patients with and without N/V is substantiated by the fact that the groups with and without N/V had similar BMI, insulin sensitivity and adiponectin levels.

The non-mechanical nausea and vomiting symptoms experienced by some patients was associated with high baseline levels of GLP-1. We hypothesise that symptoms may be ameliorated by treatment with GLP-1 inhibitors, but potential detrimental effects on weight maintenance and insulin sensitivity need to be considered. One of our patients was treated with octreotide, a somatostatin analogue that inhibits GLP-1 secretion [18], and reported improvement in N/V symptoms, with concomitant reduction in basal and post-prandial GLP-1 levels. However, octreotide also suppresses other gut hormones, such as PYY, which also decreases appetite and increases weight loss and are increased after RYGB surgery [18]. Therefore, specific GLP-1 antagonists, such as exendin 9-39, might be more beneficial in improving N/V symptoms, without interfering with the secretion of other gut peptides that may potentially affect weight loss and insulin sensitivity adversely.

This is the first report of a direct effect of GLP-1 on the secretion of an adipokine, leptin, by human adipose tissue. The chronic elevation of the gut peptide has an inhibitory effect on both systemic leptin levels and its secretion from adipose tissue. Previous reports of decreased leptin levels early after RYGB, prior to significant reduction in BMI [19], might be explained by the GLP-1-mediated inhibition of leptin. The findings in this study suggest that persistent N/V after RYGB surgery may be mediated by elevated fasting GLP-1 levels. Further studies are required to determine if treatment with specific GLP-1 antagonists ameliorates N/V symptoms without detrimental effects.
